# Serum MicroRNAs as Predictors of Diagnosis and Drug-resistance in Temporal Lobe Epilepsy: A Preliminary Study

**DOI:** 10.2174/1570159X22666240516145823

**Published:** 2024-06-25

**Authors:** Gloria Bertoli, Francesco Fortunato, Claudia Cava, Ida Manna, Francesca Gallivanone, Angelo Labate, Antonella Panio, Danilo Porro, Antonio Gambardella

**Affiliations:** 1 Institute of Molecular Bioimaging and Physiology, National Research Council (IBFM-CNR), Via F.Cervi 93, Segrate, Milan, Italy;; 2 NBFC, National Biodiversity Future Center, Palermo 90133, Italy;; 3 Institute of Neurology, Department of Medical and Surgical Sciences, University “Magna Graecia”, Germaneto, Catanzaro, Italy;; 4 Neurophysiopatology and Movement Disorders Clinic, University of Messina, Italy;; 5 IBFM-CNR, Section of Germaneto, Catanzaro, Italy;; 6 IUSS, Scuola Universitaria Superiore Pavia, Pv, Italy;; 7 Dipartimento di Biotecnologie e Bioscienze, Università degli Studi di Milano-Bicocca, Milan, Italy

**Keywords:** Temporal lobe epilepsy, TLE, microRNAs, miRNAs, diagnosis, prediction of therapy response, prognosis, circulating biomarkers

## Abstract

**Objective:**

Temporal lobe epilepsy (TLE) is the most common form of refractory focal epilepsy, and the current clinical diagnosis is based on EEG, clinical neurological history and neuroimaging findings.

**Methods:**

So far, there are no blood-based molecular biomarkers of TLE to support clinical diagnosis, despite the pathogenic mechanisms underlying TLE involving defects in the regulation of gene expression. MicroRNAs (miRNAs) have emerged as important post-transcriptional regulators of gene expression.

**Results:**

Recent studies show the feasibility of detecting miRNAs in body fluids; circulating miRNAs have emerged as potential clinical biomarkers in epilepsy, although the TLE miRNA profile needs to be addressed. Here, we analysed the diagnostic potential of 8 circulating miRNAs in sera of 52 TLE patients and 40 age- and sex-matched donor controls by RT-qPCR analyses.

**Conclusion:**

We found that miR-34a-5p, -106b-5p, -130a-3p, -146a-5p, and -19a-3p are differently expressed in TLE compared to control subjects, suggesting a diagnostic role. Furthermore, we found that miR-34a-5p, -106b-5p, -146a-5p and miR-451a could become prognostic biomarkers, being differentially expressed between drug-resistant and drug-responsive TLE subjects. Therefore, serum miRNAs are diagnostic and drug-resistance predictive molecules of TLE.

## 1 INTRODUCTION

Temporal lobe epilepsy (TLE) is the most common focal and intractable seizure disorder in adults. Despite recent advancements in its treatment, complete seizure control with antiepileptic drugs (AEDs) is achieved in only two-thirds of TLE patients [1]. Multiple pathways are responsible for intractable cases of TLE. TLE pathogenesis is thought to involve large-scale alterations in gene expression, controlling neurotransmitter signalling, ion channels, synaptic structure, neuronal death, gliosis, and inflammation. Identification of mechanisms coordinating TLE gene networks will help in identifying novel therapeutic targets and biomarkers for diagnosis, prognosis and predictors of therapy response [2]. Some TLE-associated pathways are regulated by microRNAs (miRNAs) [3-5]. Specific miRNAs have been linked to seizure-induced neuronal death or neuroprotection [6, 7]. Components of the miRNA biogenesis are altered in epileptic brain tissue, leading to synaptic alterations, cell death and inflammation. Targeting key miRNAs alters brain excitability and suppresses or exacerbates seizures, indicating the potential for miRNA-based therapeutics in epilepsy [8]. In recent years, expression profiling studies have identified over 100 different miRNAs in epileptic patients or animal models [9-12], providing evidence that epilepsy is associated with widespread changes in miRNA expression. As blood contains circulating miRNAs, TLE-associated serum miRNAs could become candidate pathology biomarkers [13-15]; whether these deregulated miRNAs are reliable for epilepsy risk prediction, diagnosis, or outcome prediction needs further verification [13, 16-18]. In order to identify circulating miRNAs as TLE biomarkers, in 2018 we developed a metanalytic approach through a Pathway Enrichment Analysis (PEA), identifying 8 TLE-associated serum miRNAs (miR-34a-5p, miR-106b-5p, miR-130a-3p, miR-146a-5p, miR-451, Let7d, miR-15a-5p, miR-19b-3p), involved in the regulation of genes controlling epilepsy hallmarks [3]. The aim of the present study was to assess the potential of those miRNAs as biomarkers for the TLE diagnosis analysing their expression profiles in serum samples of TLE patients. We then validated them for classification of AEDs-resistant vs AEDs-responsive patients.

## 2 MATERIALS AND METHODS

### 2.1 Patients’ Studies

Our case-control study was performed on 52 Mesial Temporal Lobe Epileptic (MTLE) patients (24 males and 28 females) with a mean age of 43.04 ± 18.27 years. Forty matched healthy controls (HC; 24 males and 16 females), with a mean age of 47.94 ± 9.81 years, were unrelated individuals with no neurological or psychiatric disease and no history of seizures; they voluntarily agreed to donate serum samples to our study. Our patients were consecutively recruited among those referred to the Institute of Neurology of the University of Catanzaro, Italy. All patients were from Italy and of Caucasian ethnicity. Informed consent was signed by all subjects, and our study was approved by the Institute of Neurology medical ethics committee (Protocol No. 123 on May 14, 2015) and carried out in accordance with the Declaration of Helsinki guidelines. Diagnosis of MTLE was made by two independent neurologists with special expertise in epilepsy (F.F. and A.G.), based on typical seizure semiology and electroencephalographic findings. Typical seizure semiology indicates patients experiencing focal aware or impaired awareness seizures with semiological features referable to mesial temporal lobe networks (*e.g*. rising epigastric aura, abdominal discomfort, déjà vu or jamais vu, fear, olfactory or gustatory), according to the latest statements by ILAE Task Force for definition of epilepsy syndromes [19]. All patients recruited also performed an awake routine video-EEG with supplementary T1 and T2 electrodes. Typical interictal electroencephalographic findings include epileptic abnormalities (focal spikes or sharp waves) involving temporal regions, as they occurred over electrodes F7, F8, T3, T4, T1, and T2. The localization of epileptiform abnormalities (EA) was based on the site of maximum voltage on referential montage or phase reversal on bipolar montage. Any suggestion of seizure onset outside the mesial temporal structures by semiology or EEG findings was an exclusion criterion. All patients underwent brain magnetic resonance imaging (MRI) using a 3-Tesla GE MR750 scanner (GE Healthcare, Rahway, NJ, USA), according to the HARNESS-MRI protocol [20]. Patients were excluded if they had an MRI-visible lesion (due to stroke, head trauma, malformations of cortical development or tumors) other than hippocampal sclerosis. Hippocampal sclerosis was diagnosed according to established typical features on MRI (*i.e*., a T2-weighted or fluid-attenuated inversion recovery scan). Other exclusion criteria were severe organ diseases, progressive neurological diseases, autoimmune diseases, mental disorders, diabetes, or major congenital neurological diseases; patients with abnormal blood routine examination. Clinical and demographic features of MTLE patients recruited are summarized in Table **1**. Patients were subsequently divided into two groups according to their response to anti-seizure medications (ASMs) treatment: 36 drug-responsive and 16 drug-resistant patients. Drug resistant patients were defined by ILAE’s definition: failure of adequate administration of two ASMs at optimal doses [21]. Since this study is a validation study to define the role of a subset of defined miRNAs on a limited retrospective cohort of epileptic patients, an a-posterior calculation of sample size required to have sufficient statistical power was performed, following [22], in order to support the significance of the results of this case study. Data by Wang *et al*. [23] were used to calculate the variance and fraction of non-differentially expressed miRNAs. The size.fdr R package was used for the sample size calculation. A summary scheme of the approach proposed in this paper is provided in Fig. (**S1**).

### 2.2 Reverse Transcription and Real-time Quantitative PCR (RT-qPCR)

Six ml of whole blood of each participant was collected, fasting, and processed for standard serum isolation. Anonymized cleared serum samples were isolated and stored at −80°C until use. RNA was isolated from 200ul of serum by Serum/Plasma miRNeasy kit (Qiagen). Serum RNA was reverse transcribed by MystiCq microRNA cDNA Synthesis Mix kit (Sigma). The cDNA was the template for SYBR Green-based real-time quantitative PCR (RT-qPCR) analysis, using the kit spike-in as a reference. MiRNAs were amplified with specific primers, whose sequences are disposable upon request. The level of expression of each miRNA was reported in the graphs as relative expression (2^-DCt) in TLE serum samples compared to healthy serum samples [24]. T-test analysis was applied, and the p-value was calculated (indicated in the text).

### 2.3 *In silico* Validation of TLE-associated miRNA Signature

For the validation of signature performance in the classification process, we developed a support vector machine (SVM) using the R-package e1071. We optimize the SVM feasible learning parameters with a kernel type = linear (see e1071 documentation at [25] (https://CRAN.R-project.org/package=e1071]). We implemented a cross-validation method, which allows randomly assigning half of the original dataset for the training set, and half of the dataset to the testing set [26]. This implemented SVM method was applied on GSE114697 independent GEO dataset. This dataset contains miRNA profiles of 16 healthy subjects and 16 TLE patient serum samples, both male and female, identified by continuous video-EEG recording, in two different countries [27]. We selected miRNA expression levels of the 16 TLE patients before seizure, as the increase in the inflammatory status of the patients due to the sudden seizure could alter the expression of other miRNAs not associated to TLE. The performance of the proposed classification algorithm was evaluated with accuracy, specificity and sensitivity. In order to identify the most important miRNAs for the classification of TLE vs. healthy controls (HC), we considered the miRNA expression levels in all the possible combinations.

### 2.4 *In silico* Analysis of Potential miRNA Targets

SpidermiR software [28] allows the identification of the predicted targets of miR-34a-5p, 106b-5p, -130a-3p, -146a-5p, miR-19b, considering miRNA targets from DIANA [29], miRanda [30], PicTar [31], TargetScan [32] and miRDB [33] predictive algorithms. We considered the mRNAs targets of the miRNAs only if they are present in at least 2 of the 5 algorithms. In this phase of the research, we didn’t perform the *in vitro* validation of the proposed targets. As the identified target network contains several possible direct targets, we will investigate in the future the direct interactor mRNA of each miRNA to be tested *in vitro*. Pathway analysis was carried out with the R-package clusterProfiler [34]. We identified pathways enriched with miRNA targets using KEGG pathways (https://www.genome.jp/kegg/) and a statistical test based on the hypergeometric distribution.

## 3 RESULTS

### 3.1 miRNAs’ Selection

Based on the high-throughput discovery study of Wang *et al*. [23], a common standard deviation of 0.68 and a proportion of non-differentially expressed miRNAs of 0.98 were considered for power analysis. Using these data to detect a 2-fold change in miRNA expression levels, a sample size of 19 subjects was estimated for achieving at least 80% of statistical power with a false discovery rate of 10% and an estimated proportion of non-differentially expressed miRNAs of 0.98. The fold change in expression levels obtained by Wang [23] is greater than 2 for all the considered TLE-altered miRNAs, except for miR-15a-5p, which was thus excluded from further analysis. MiR-34a-5p, miR-106b, miR-130a-3p, miR-146a-5p, miR-451a-5p, Let7d-5p and miR-19b-3p were subsequently validated in studies on serum samples from 52 TLE (see Table **1** for clinical characteristics) and 40 healthy volunteers.

### 3.2 Diagnostic Potential of Human Circulating miRNAs

**Fig. (1):**
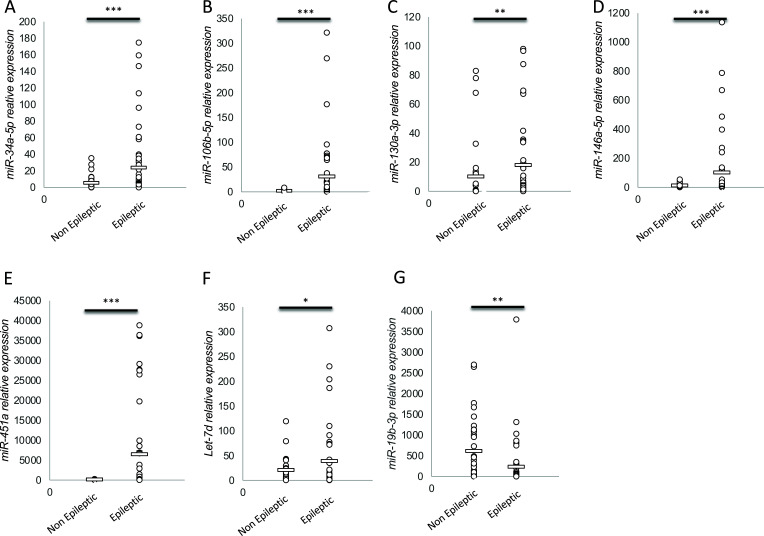
RT-qPCR analysis of 7 diagnostic serum miRNAs. Scattered plots represent miR-34a-5p (**A**), miR-106b-5p (**B**), miR-130a-3p (**C**), miR-146a-5p (**D**), miR-451 (**E**), Let-7d (**F**), miR-19b-3p (**G**) relative expression (2^-DCT) in Non-epileptic *versus* TLE epileptic patients (*t*-test, *p*-value < 0.05, *; <0.01,**; NS, not significant). Average values are indicated by the bar.

*In vitro* Real-Time -quantitative PCR (RT-qPCR) analysis revealed 5/7 circulating miRNAs differentially expressed among TLE patients and healthy subjects (Fig. **1**), confirming the *in silico* prediction [3]: miR-34a-5p (t-test, *p*-value = 0.017, Fig. **1A**), -106b-5p (t-test, *p*-value = 0.050, Fig. **1B**), -130a-3p (t-test, *p*-value = 0.045, Fig. **1C**), -146a-5p (t-test, *p*-value = 0.008, Fig. **1D**) were significantly upregulated, while miR-19b-3p was significantly downregulated (t-test, *p*-value = 0.009, Fig.**1G**). Alterations of miR-451a (Fig. **1E**) and let7d (Fig. **1F**) were not statistically significant.

To be sure of the diagnostic ability of the five circulating miRNAs, the *in silico* validation was performed on GSE114697 independent dataset, which contains all the miRNAs considered in our analysis, except miR-34a. With this bioinformatics classification, we then validated 4 miRNAs for their diagnostic ability: miRNA-19b, miRNA-106b, miRNA-130a, and miRNA-146a.

The accuracy of classification for epileptic *versus* non-epileptic (control subjects made with only a single miRNA on GSE114697) was lower than 68%, with sensitivity and specificity values of 37-75% and 25-62%, respectively (Table **2**).

The best predictor for epileptic *versus* non-epileptic classification was the combination of miRNA-19b, miRNA-106b, miRNA-130a, and miRNA-146a, as they collectively achieved a good performance (accuracy, sensitivity and specificity > 0.60).

### 3.3 TLE-associated miRNAs and Target Genes Analysis

**Fig. (2):**
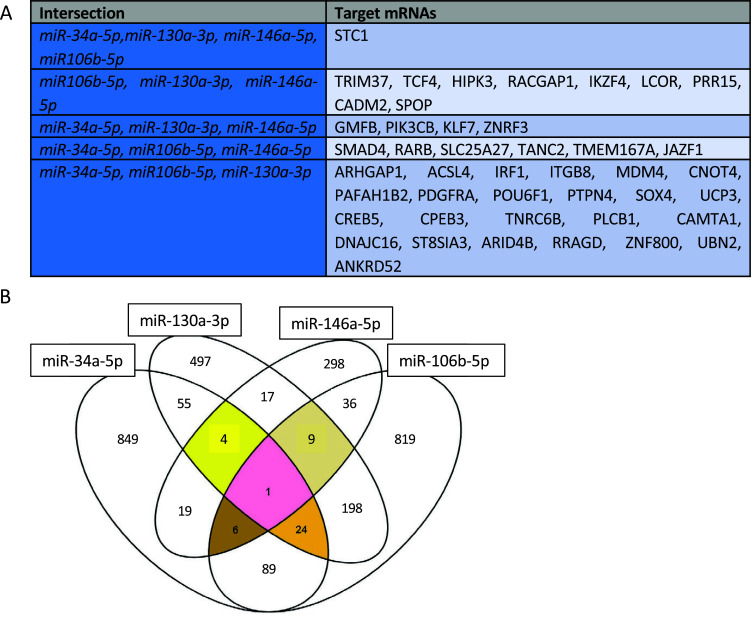
Analysis of miRNA predicted target. (**A**) Summary table of the predicted target mRNAs of upregulated miR34a-5p, miR-130a-3p, miR-146a-5p and miR-106b-5p. (**B**) Diagram representing common target mRNAs among miR-34a-5p, -130a-3p, -146a-5p, and -106b-5p in TLE.

**Fig. (3):**
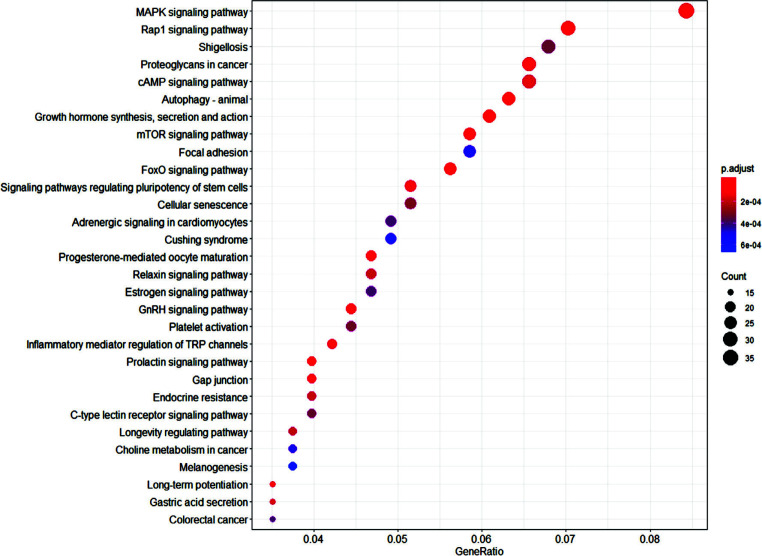
Pathways enriched with miR-19b-3p targets. The size of the circles indicates the number of miRNA targets in that pathway. The colour of the circles shows the adjusted *p*-values.

A summary table of SpidermiR results for upregulated miRNAs is shown (Fig. **2A**). The intersection between the lists of candidate target genes for each up-regulated miRNA (Fig. **2B**) revealed that among 1047 miR-34a-5p-target mRNAs, 120 are in common with miR-106a-5p, 84 with miR-130a-3p, 30 with miR-146a-5p; of the 805 target mRNAs of miR-130a-3p, 232 are in common with miR-106b-5p and 31 with miR-146a-5p; of the 1182 predicted target of miR-106b-5p, 52 are in common with miR-146a-5p. Five targets are shared between miR-34a-5p, miR-130a-3p and miR-146a-5p; ten mRNAs are shared between miR-146a-5p, miR-130a-3p and miR-106b-5p; twenty-five mRNAs are the possible target of miR34a-5p, miR-106b-3p and miR-130a-3p. Only Stanniocalcin 1 (STC1) is possibly the target of all four upregulated miRNAs. We found 1058 potential targets for miR-19b-3p with SpidermiR. On the biological role of these targets, we found several mechanisms involved in the maintenance of the excitatory/inhibitory balance, MAPK signaling pathways and autophagy (Fig. **3**).

On the biological role of these targets, we found several mechanisms involved in the maintenance of the excitatory/inhibitory balance, MAPK signaling pathways and autophagy (Fig. **3**).

### 3.4 Five miRNAs are Differentially Expressed between Drug-responsive and Drug-resistant TLE Subjects

**Fig. (4):**
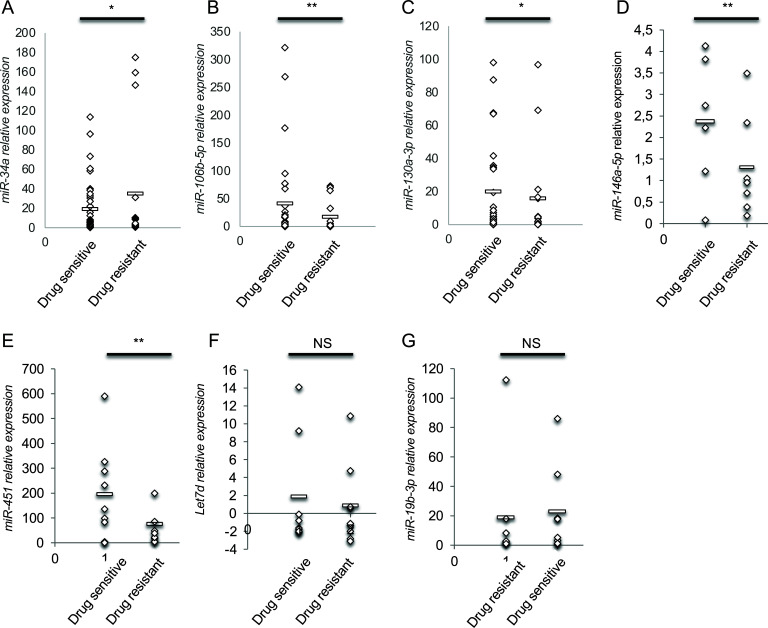
Relative expression of 6 serum miRNAs predictors of AED response. Scattered plots represent miR-34a-5p (**A**), miR-106b-5p (**B**), miR-146a-5p (**C**), miR-451 (**D**), Let-7d (**E**), miR-19b-3p (**F**-**G**) expression in AEDs drug-responsive *versus* drug-resistant TLE subjects (t-test, *p*-value < 0.05,*; < 0.001,***). Average values are indicated by the bar.

Despite the limitation due to the reduced number of TLE patients, a pilot study was conducted to identify if miRNAs are also able to predict the drug sensitivity of a TLE patient by dividing the 21 TLE sera into drug-responsive and drug-resistant TLE sera. We analysed the expression levels of the 7 circulating miRNAs for which the biological sample size was sufficient on the basis of power analysis. For this reason, we excluded miR-130a-3p from further analysis. We identified circulating miR-34a-5p as upregulated miRNA (t-test, *p*-value = 0.027) and miR-106b-5p (t-test, *p*-value = 0.0032), miR-130a-3p (t-test, *p*-value = 0.011), miR-146a-5p (t-test, *p*-value = 0.009), and miR-451 (t-test, *p*-value = 0.009) significantly downregulated miRNAs in Drug-resistant serum samples (Figs. **4B**-**D**); all these miRNAs could be considered predictors of response to therapy treatment and their loss could sustain the development of therapy resistance. Unfortunately, no public GEO dataset containing drug-responsive and drug-resistant epileptic sera miRNA profile is publicly available to perform an *in silico* validation (September 2021). In order to identify the molecular mechanisms targets of the downregulated miRNAs, SpidermiR software was applied [28]. MiR-146a-5p and miR-451 have 14 mRNAs as putative common targets, mainly involved in cell cycle regulation, although no common biological pathway emerged from String analysis.

## 4 DISCUSSION

### 4.1 Diagnostic Serum miRNAs in mTLE

In this study, we showed that the upregulated expression of miR-34a-5p, -106b-5p, -130a-3p, 146a-5p, and the downregulated expression miR-19a-3p in serum samples are able to distinguish TLE patients from healthy subjects. Some of these miRNAs have already been proposed as diagnostic miRNAs in other papers by our laboratory, such as miR-146a [35] or miR-34a [3]. In both publications, we selected diagnostic circulating miRNAs by analysing those already published in the literature, not performing bioinformatic analysis on public databases or any validation analysis on an independent dataset for their classification ability. In this case, the good classification performance of miRNA-19b-3p, miRNA-106b, miRNA-130a, and miRNA-146a combined signature on the GSE114697 dataset, although the classification performance is not great (possibly due to the reduced samples size of the dataset), confirmed that this signature is able to correctly identify TLE form healthy cases. The result is particularly important as this 4-miRNA signature has been validated in sera of TLE patients, thus with a low invasive method. Circulating brain-enriched miRNAs have already been proposed as novel biomarkers for the differential diagnosis of neurodegenerative disorders, such as Alzheimer’s disease, frontotemporal dementia, Parkinson’s disease and amyotrophic lateral sclerosis [36]. The analysis described in this manuscript refers to a set of miRNAs that was identified in 2018 with a metanalytic approach [3]. Other studies proposed the use of different sets of miRNAs for different purposes, such as those proposed as possible markers of TLE outcome after surgical treatment [37] or starting by a different initial biological material, such as sclerosis-associated mTLE hippocampal tissue [38], or considering only those miRNAs associated to a specific pathway (*i.e*., neuroinflammation [39] or ion channels [40]). Differences between miRNAs found in our analysis and other studies could be due also to the age of the epileptic patients: for example, we expect that in children, other miRNAs could be altered for adults [41-43] as some of them could have a role also in the growth of the child. Differences in blood miRNA profiles have been observed in two groups of healthy children, newborns compared to age 7 children [44]. The differences in the selection of diagnostic miRNA could also be due to a different type of identification method, such as microarray [45], RNAseq [46] or OpenArray platform [47]. The analysis of the possible mRNA targets of miRNA-19b, miR-34a, miRNA-106b, miRNA-130a, miRNA-146a could suggest TLE-associated cellular functions. MiR-34a upregulation has been found to be a regulator of apoptosis in TLE hippocampal neurons [9-11]. Notably, miR-130a-3p, miR-106b-5p and miR-146a-5p were upregulated in sera of epileptic patients [23], being possibly associated with neuroinflammation status (*i.e*. [48]). Only STC1 is a common target of miR-34a-5p, 106b-5p, -130a-3p, -146a-5p. This gene encodes for a glycoprotein hormone involved in calcium/phosphate homeostasis [49]. The downregulation of STC1 protein, due to miRNAs upregulation, could contribute to the hypercalcemic neurotoxicity and cell death observed in the TLE hippocampus [49]. Some calcium blockers have been proposed as possible therapeutic agents in epilepsy [50]. Moreover, miR-34a, miR-106b-5p, and miR-146 could have an active role in neurogenesis, cell cycle control, and cell proliferation [3]. Regarding the downregulated miR-19b-3p, it is enriched in adult hippocampal neural progenitor cells and involved in the control of neuronal cell migration from the subgranular zone of the hippocampal dentate gyrus [51, 52]. The misguidance of mossy fibers, including axonal branching and reverse projection, is a TLE pathological hallmark [53, 54]. Moreover, miR-19b decrease could lead to neuronal loss in the dentate gyrus of the hippocampus. Several mRNAs, targets of miR-19b-3p, are involved in the modulation of neurotransmission, or MAPK signaling pathways, resulting in hyperexcitability due to ion channels and receptors excessive synthesis [55], function already analysed recently [40, 56]. In epilepsy, senescence has a main role, leading to increased secretion of pro-inflammatory cytokines [39], matrix metalloprotease and growth stimulating factors [56]. This process leads to microglia activation and infiltration of leukocytes in TLE sclerotic tissue [57]. Also, autophagy, sustained by the loss of miR-19b-3p expression, has been described in epilepsy as a degradative pathophysiological process [58].

### 4.2 Predictive Serum miRNAs in Drug-resistant mTLE

We found a significant increase in miR-34a-5p and a decreased expression of miR-106b-5p, -130a-3p, -146a-5p and -451a in AEDs-resistant TLE subjects. Looking at common target mRNAs among downregulated miRNAs, we found 14 shared mRNAs (GDNF, PDE6H, PHKB, PPEF1, TGIF1, EIF1AY, PRDX4, NUDT5, SEC23IP, TTC39A, CLASP2, TNFAIP8, OR56B4, and RAB41), but we were unable to find out a single KEGG pathway including all these mRNAs. Many of these genes belong to the purine metabolism pathways [59]. Purine adenosine is an endogenous anticonvulsant, effective in numerous seizure models and in pharmacoresistant kainate-induced seizures [60]. In TLE subjects, we confirmed miR-146a-5p upregulation, as described in the literature (*i.e*. [61]), but we observed a significant decrease in its expression in drug-resistant patients’ sera [62]. We could hypothesize that in the TLE onset, miR-146a-5p increase maintains silenced mRNAs of the starting phase of TLE, but in the advanced stage, its decrease is responsible for drug-resistant development. In this view, the endoplasmic reticulum-lysosome-Golgi network and intracellular vesicle trafficking could be important in AEDs resistance development [63, 64].

## CONCLUSION

The main discoveries in our work are 1. the validation of miR-34a-5p, 106b-5p, -130a-3p, -146a-5p and miR-19a-3p miRNAs as diagnostic molecules; 2. the explanation of their possible functions by *in silico* identification of their molecular targets; 3. the identification of miR-34a-5p, -106b-5p, -146a-5p and miR-451a as potential biomarkers for drug-resistant epilepsy. The identified miRNAs are important biomarkers of TLE to be easily analysed in patients’ serum and could also help in the identification of intractable cases of TLE.

## Figures and Tables

**Table 1:** Demographic data and clinical characteristics of the patients and controls (T-test p values are indicated). Abbreviations: SD, standard deviation, yrs, years, AED, antiepileptic drug, na, not applicable.

**-**	**TLE**	**HC**	***p* value**
No.	52	40	-
Gender (male/female)	24/28	24/16	*p* > 0.05
Age, mean ± SD (yrs)	43.04 ± 18.27	47.94 ± 9.81	*p* > 0.05
Age of onset, mean ± SD (yrs)	24.47 ± 19.65	na	-
Disease duration, mean ± SD (yrs)	18.57 ± 17.38	na	-
Family history of epilepsy, N (%) Yes No	18 (36%) 32 (64%)	na	-
Seizure frequency, N (%) Daily Weekly Monthly	7 (14%) 12 (24%) 31 (62%)	na	-
EEG, N (%) Bilateral Left Right	10 (19,61%) 24 (47,05%) 17 (33,34%)	na	-
Hippocampal Sclerosis, N (%) Bilateral Left Right No Sclerosis	1 (2%) 3 (6%) 6 (12%) 40 (80%)	na	-
Febrile Seizures, N (%) Yes No	13 (26%) 37 (74%)	na	-
Focal to Bilateral Tonic-Clonic Seizures, N (%) Yes No	42 (84%) 12 (24%)	-	-
AED therapy at the last clinic visit, N (%) Monotherapy Polytherapy	21 (40,39%) 31 (59,61%)	na	-
Drug-responsive, N (%)	36 (69.23%)	na	-
Drug-resistant, N (%)	16 (30.77%)	na	-

**Table 2:** Performance of classification TLE *vs*. control in GSE114697 dataset.

***Single or Combination of miRNAs***	***ACC***	***SPEC***	***SENS***
*miRNA-19b*	0.56	0.5	0.62
*miRNA-106b*	0.5	0.75	0.25
*miRNA-130a*	0.68	0.37	1
*miRNA-146a*	0.5	0.37	0.62
*miRNA-19b, miRNA-106b*	0.5	0.75	0.25
*miRNA-19b, miRNA-130a*	0.6875	0.375	1
*miRNA-19b, miRNA-146a*	0.5	0.375	0.625
*miRNA-106b, miRNA-130a*	0.6875	0.875	0.5
*miRNA-106b, miRNA-146a*	0.5	0.625	0.375
*miRNA-130a, miRNA-146a*	0.6875	0.375	1
*miRNA-19b, miRNA-106b, miRNA-130a*	0.6875	0.875	0.5
*miRNA-19b, miRNA-106b, miRNA-146a*	0.5625	0.75	0.375
*miRNA-19b, miRNA-130a, miRNA-146a*	0.5	0.25	0.75
*miRNA-106b, miRNA-130a, miRNA-146a*	0.625	0.5	0.75

**Abbreviations:** (ACC: accuracy, SPEC: specificity, SENS: sensitivity).

## Data Availability

Not applicable.

## LIST OF ABBREVIATIONS

ACC: Accuracy
AEDs: Anti-epileptic Drugs
miRNA: microRNA
MRI: Magnetic Resonance Imaging
mTLE: Mesial Temporal Lobe Epilepsy
Sens: Sensitivity
SPEC: Specificity
