# Supplementary material for: Serum MicroRNAs as Predictors of Diagnosis and Drug-resistance in Temporal Lobe Epilepsy: A Preliminary Study
Source: Curr Neuropharmacol. 2024 Jun 25;22(14):2422–32. doi: 10.2174/1570159X22666240516145823 (PMC11451323; doi:10.2174/1570159X22666240516145823)
Supplement: Supplementary file 1 [file CN-22-2422_SD1.pdf]

Supplementary Material

Serum MicroRNAs as Predictors of Diagnosis and Drug-resistance in Temporal Lobe Epilepsy: A Preliminary Study

Gloria Bertoli[1,2,*,#], Francesco Fortunato[3,#], Claudia Cava[1,2,6], Ida Manna[5,*], Francesca Gallivanone[1,2], Angelo Labate[4], Antonella Panio[1], Danilo Porro[1,2,7] and Antonio Gambardella[1,3,*]

[1]Institute of Molecular Bioimaging and Physiology, National Research Council (IBFM-CNR), Via F.Cervi 93, Segrate, Milan, Italy; [2]NBFC, National Biodiversity Future Center, Palermo 90133, Italy; [3]Institute of Neurology, Department of Medical and Surgical Sciences, University "Magna Graecia", Germaneto, Catanzaro, Italy; [4]Neurophysiopatology and Movement Disorders Clinic, University of Messina, Italy; [5]IBFM-CNR, Section of Germaneto, Catanzaro, Italy; [6]IUSS, Scuola Universitaria Superiore Pavia, Pv, Italy; [7]Dipartimento di Biotecnologie e Bioscienze, Università degli Studi di Milano-Bicocca, Milan, Italy

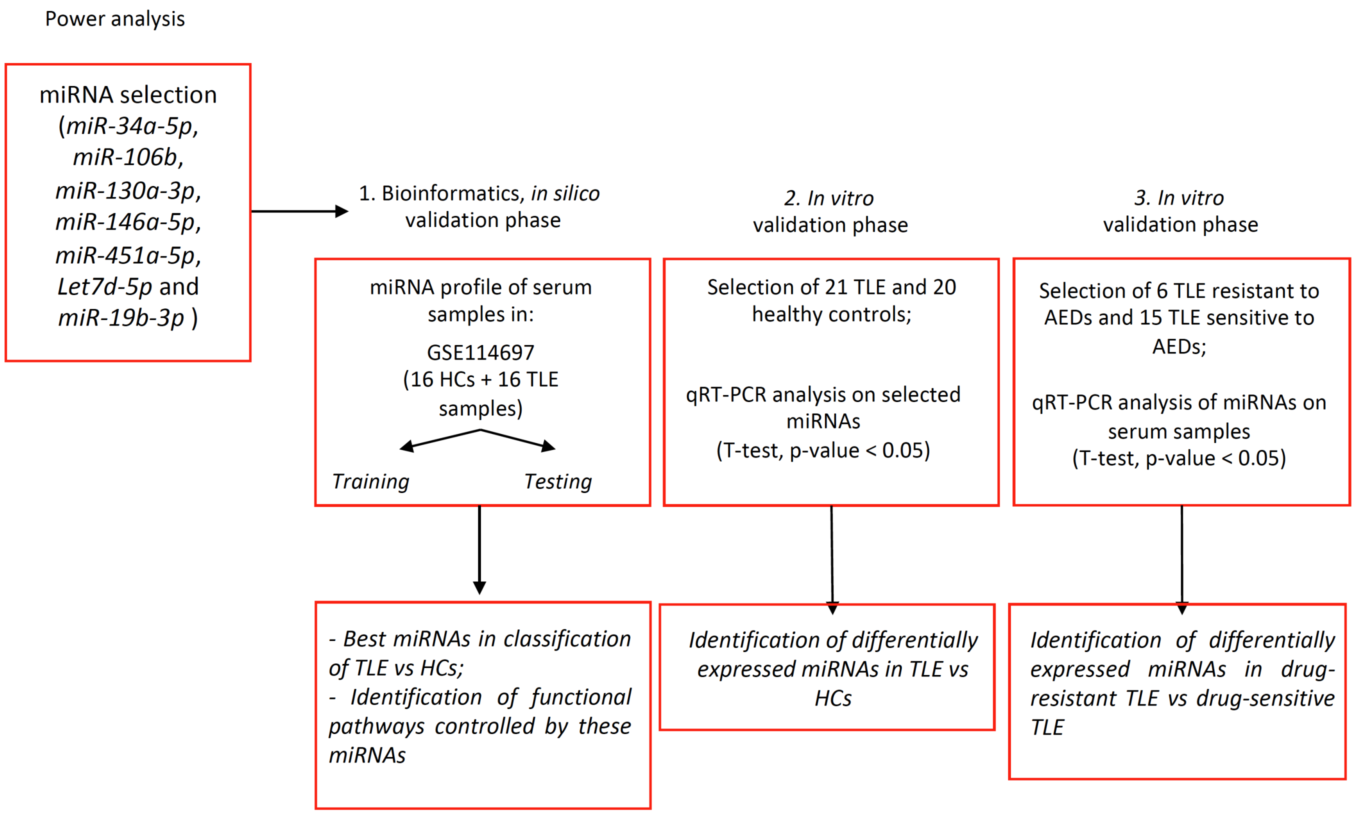

1875-6190/24 $65.00+.00 © 2024 Bentham Science Publishers
